# Supplementary material for: Mice with renal-specific alterations of stem cell-associated signaling develop symptoms of chronic kidney disease but surprisingly no tumors
Source: PLoS One. 2024 Mar 21;19(3):e0282938. doi: 10.1371/journal.pone.0282938 (PMC10957084; doi:10.1371/journal.pone.0282938)
Supplement: S2 Fig — (PDF) [file pone.0282938.s002.pdf]

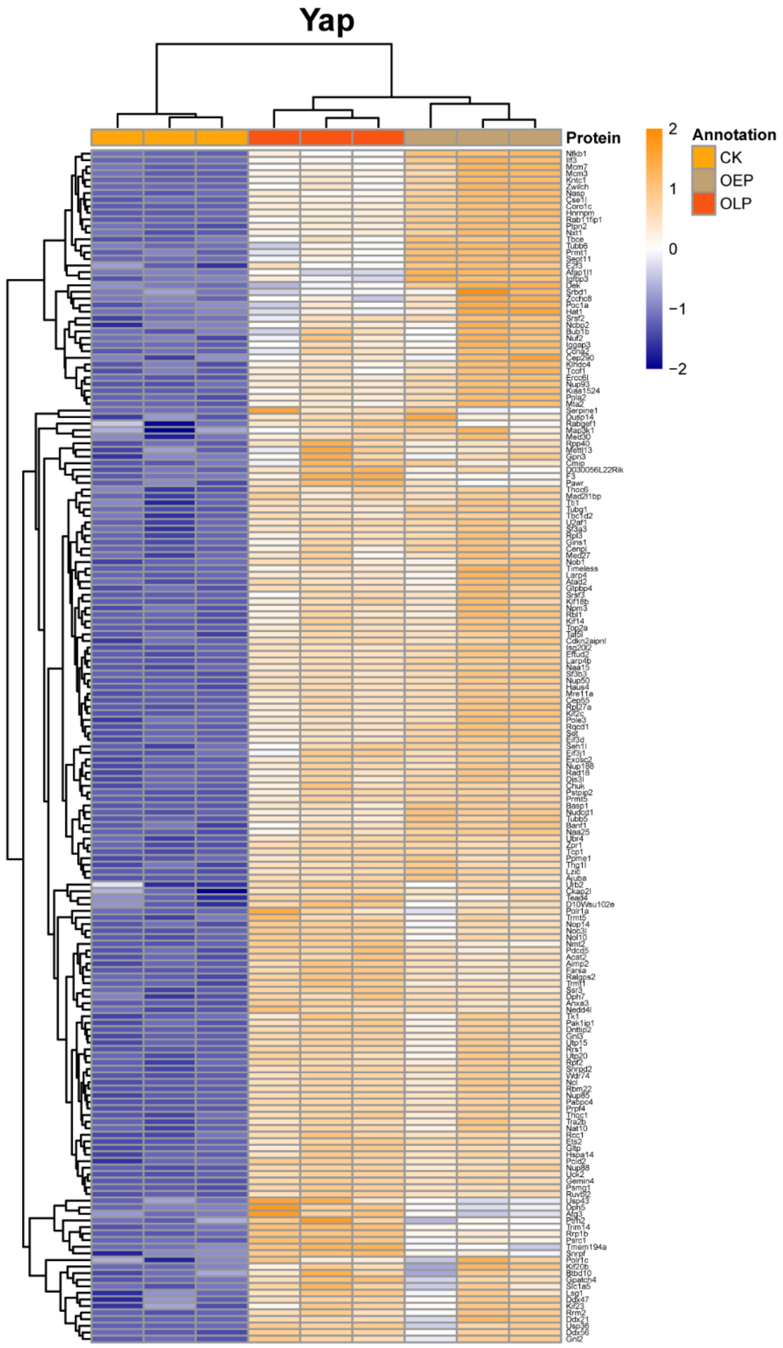

S2 Fig. Yap signaling is upregulated in kidney tubuloids. Proteomic heatmap for direct Yap targets in both early (OEP) and long-term (OLP) passage tubuloids in comparison to mouse kidney epithelia (control kidney, CK). Data information: the heatmap shows normalized log2 intensity values for three independent replicates of CK, OEP and OLP. A 5% FDR (adjusted P-value < 0.05) cutoff and a log2 fold change cutoff of > 0 were applied for both OEP over CK and OLP over CK. Values were scaled (z-score by row) with breaks from ≤ -2 to ≥ 2.
