# Supplementary material for: Mice with renal-specific alterations of stem cell-associated signaling develop symptoms of chronic kidney disease but surprisingly no tumors
Source: PLoS One. 2024 Mar 21;19(3):e0282938. doi: 10.1371/journal.pone.0282938 (PMC10957084; doi:10.1371/journal.pone.0282938)
Supplement: S3 Fig — (PDF) [file pone.0282938.s003.pdf]

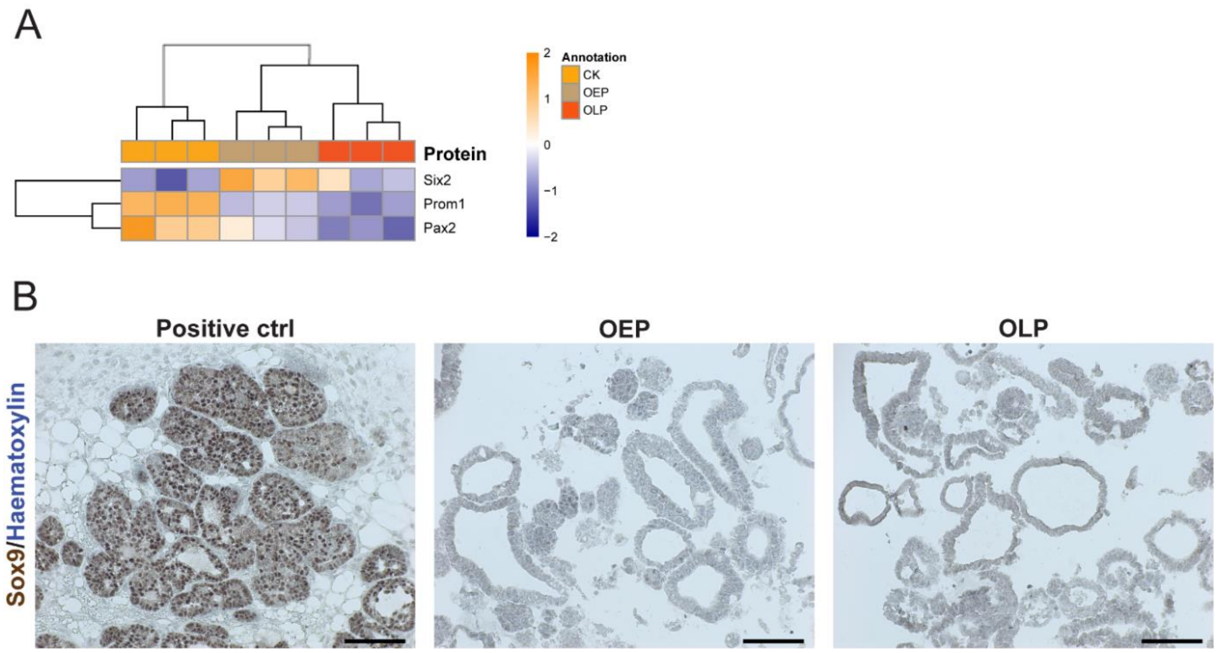

S3 Fig. Expression of selected markers of resident stem/progenitor cells in tubuloids. (A) Proteomic heatmap for Six2, Prom1 and Pax2 in both early (OEP) and long-term (OLP) passage tubuloids in comparison to freshly isolated Epcam-positive kidney epithelial cells (control kidney, CK). (B) Immunohistochemistry for Sox9 in both early (OEP) and long-term (OLP) passage tubuloids. Data information: in A, the heatmap shows normalized log2 intensity values for three independent replicates of CK, OEP and OLP. A 5% FDR (adjusted P-value < 0.05) cutoff and a log2 fold change cutoff of > 0 were applied for both OEP over CK and OLP over CK. For Six2, OLP over CK values were not significant in a pairwise comparison. The values were scaled (z-score by row) with breaks from ≤ -2 to ≥ 2. Scale bars in B, 100 µm. A positive ctrl used, high-Wnt and high-Met mouse mammary gland tumors. Nuclei are counterstained with haematoxylin. One independent replicate per condition was examined.
