# Supplementary material for: Mice with renal-specific alterations of stem cell-associated signaling develop symptoms of chronic kidney disease but surprisingly no tumors
Source: PLoS One. 2024 Mar 21;19(3):e0282938. doi: 10.1371/journal.pone.0282938 (PMC10957084; doi:10.1371/journal.pone.0282938)
Supplement: S4 Fig — (PDF) [file pone.0282938.s004.pdf]

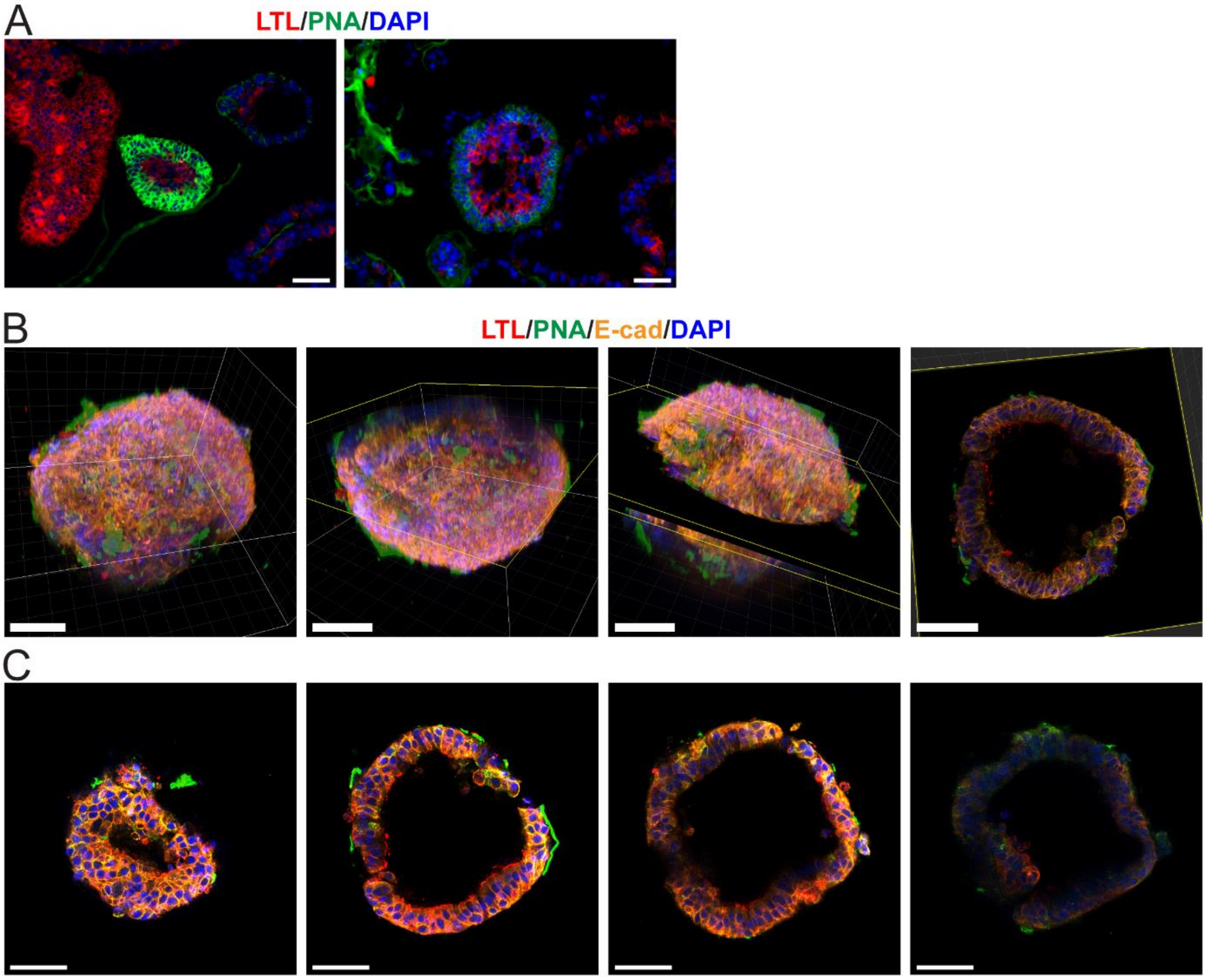

S4 Fig. Markers of tubular epithelial cells are expressed in 3D-reconstructed kidney tubuloids. (A) 2D immunofluorescence of a solid (left) and an alveolar (right) tubuloid positive for both LTL (red) and PNA (green). (B and C) Whole-mount 3D confocal microscopy of a cystic tubuloid positive for LTL (red), PNA (green) and E-cad (orange). (B) A 3D reconstruction of z-stacks of a cystic tubuloid. From left to right: a 3D-reconstructed tubuloid seen from the top-side (coordinates given for orientation), top-side view opened by a clipping plane, top-side view with a marked orthogonal slice (8 µm) and extended orthogonal slice (8 µm) only. (C) Selected 2D images of z-stacks at different depths (top, middle, bottom) of a cystic tubuloid. Data information: scale bars, 50 µm. Nuclei are counterstained with DAPI. Three independent replicates were examined.
