# Supplementary material for: Mice with renal-specific alterations of stem cell-associated signaling develop symptoms of chronic kidney disease but surprisingly no tumors
Source: PLoS One. 2024 Mar 21;19(3):e0282938. doi: 10.1371/journal.pone.0282938 (PMC10957084; doi:10.1371/journal.pone.0282938)
Supplement: S5 Fig — (PDF) [file pone.0282938.s005.pdf]

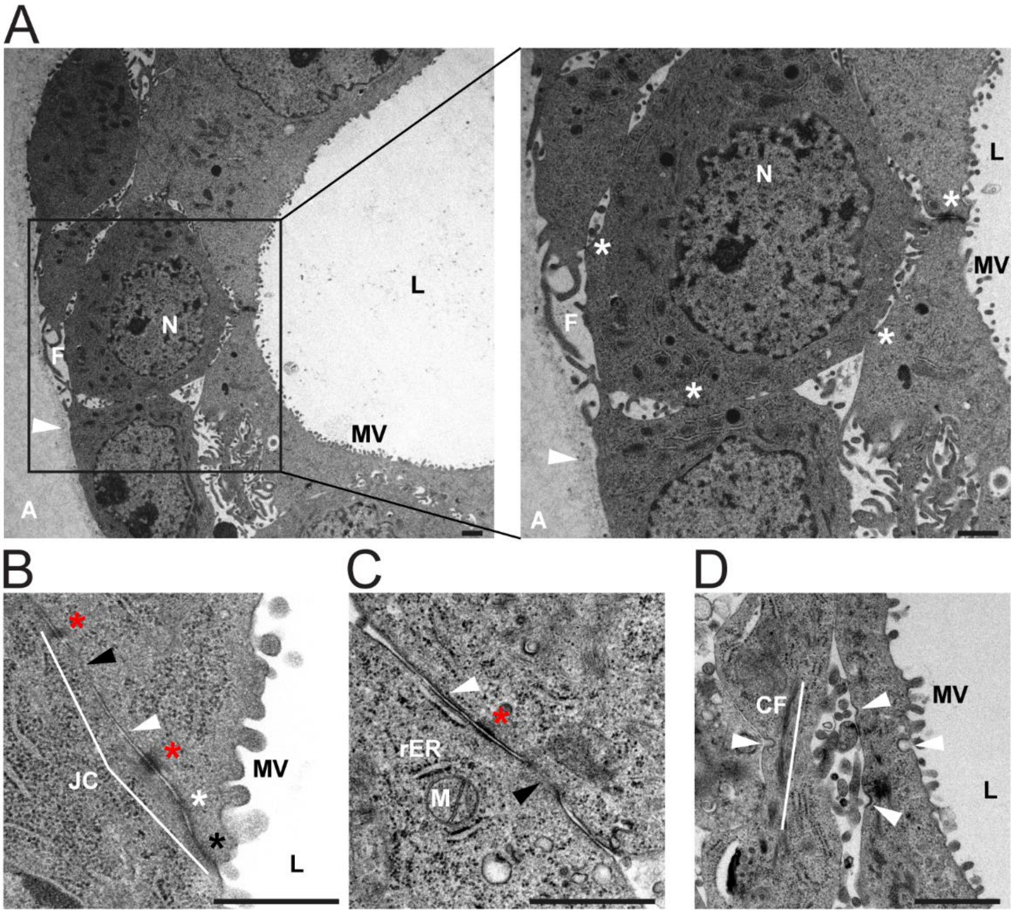

S5 Fig. Kidney tubuloids display tubular epithelial polarity and complexity. (A) Transmission electron microscopy of a cystic tubuloid with visible microvilli (MV) at the luminal side (L), a basal lamina without MV at the opposite basal side (arrowhead), filopodia (F), cell-cell contacts (asterisks) and surrounding agarose (A). (B) Detailed view showing an epithelial junctional complex (JC) between lateral membranes of neighboring cells. Shown are a tight junction (zonula occludens, black asterisk), an adherens junction (belt desmosome, zonula adhaerens, white asterisk), spot desmosomes (macula adhaerens, red asterisks) and closely aligned or nearly fused lateral membranes (white and black arrowhead, respectively). (C) Detailed view of a cell at the basal side. Shown are a spot desmosome (red asterisk) and closely aligned or nearly fused lateral membranes (white and black arrowhead, respectively). (D) Detailed view of endocytic events at apical and lateral membranes with clathrin-coated pits (diameter of around 100 nm, arrowheads). Data information: scale bars, 1 µm. Abbreviations; N, nucleus; M, mitochondrion; rER, rough endoplasmatic reticulum (dark ordered dots); CF, cytoskeleton filaments (actin, dark structures along the white line). In A, insets are enlarged on the right. Three independent replicates were examined.
