# Supplementary material for: Mice with renal-specific alterations of stem cell-associated signaling develop symptoms of chronic kidney disease but surprisingly no tumors
Source: PLoS One. 2024 Mar 21;19(3):e0282938. doi: 10.1371/journal.pone.0282938 (PMC10957084; doi:10.1371/journal.pone.0282938)
Supplement: S6 Fig — (PDF) [file pone.0282938.s006.pdf]

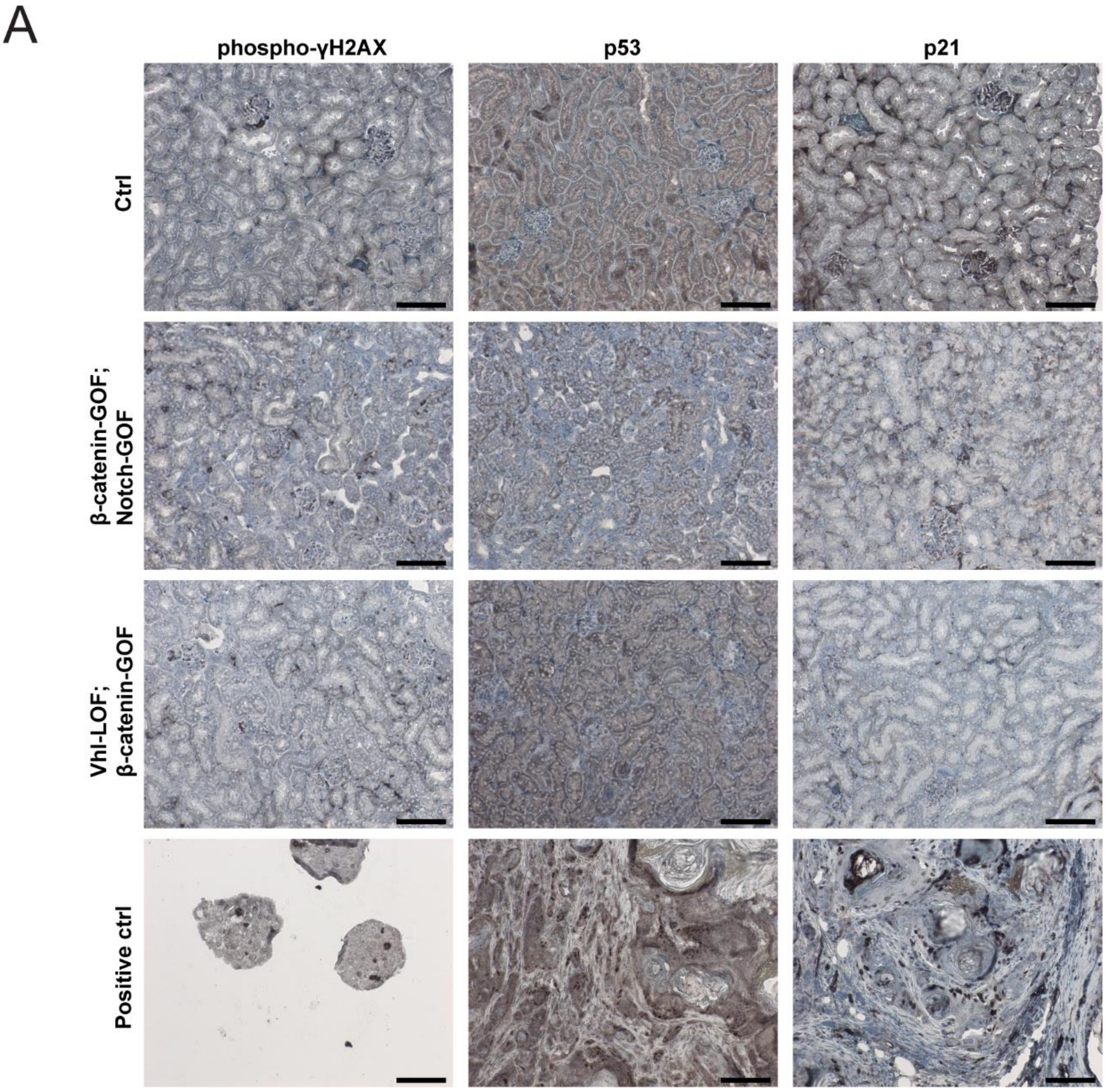
A
phospho-γH2AX
p53
p21
Ctrl
β-catenin-GOF; Notch-GOF
Vhl-LOF; β-catenin-GOF
Positive ctrl

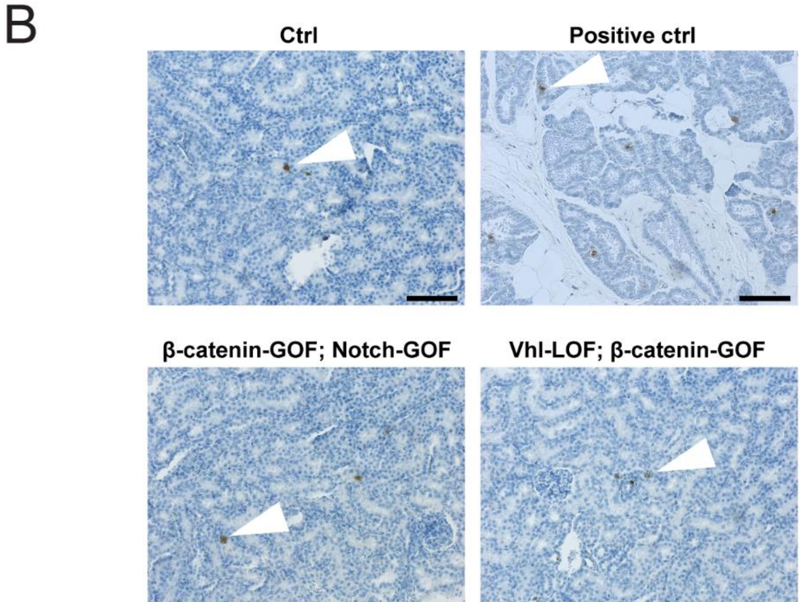
B
Ctrl
Positive ctrl
β-catenin-GOF; Notch-GOF
Vhl-LOF; β-catenin-GOF

S6 Fig. No DNA damage, growth arrest or senescence and increased apoptosis was observed in mutant kidneys. (A) Immunohistochemistry for nuclear phospho-γH2AX (left panel), p53 (middle panel) and p21 (right panel) in β-catenin-GOF; Notch-GOF and Vhl-LOF; β-catenin-GOF mutant cortical kidneys versus controls. (B) Immunohistochemistry for blunt ends of double-stranded DNA breaks in apoptotic cells (TUNEL assay) in β-catenin-GOF; Notch-GOF and Vhl-LOF; β-catenin-GOF mutant cortical kidneys versus controls. Data information: scale bars in A, 100 µm. Positive controls used; in the left panel, non-adherent spheres derived from a human colorectal carcinoma cell line LS174T with doxycycline-induced shRNA-mediated knockdown of *MLL1*; in the middle and right panel, mouse Pi3k-GOF; β-catenin-GOF; p53-GOF mammary gland tumors. Nuclei are counterstained with haematoxylin. Three independent replicates per line were examined. Scale bars in B, 100 µm. A positive control used (included in the kit), normal rat mammary gland 3-5 days after weaning of pups. Nuclei positive for blunt ends are marked by arrowheads. Nuclei are counterstained with haematoxylin. Three independent replicates per line were examined.
