# Supplementary material for: Mice with renal-specific alterations of stem cell-associated signaling develop symptoms of chronic kidney disease but surprisingly no tumors
Source: PLoS One. 2024 Mar 21;19(3):e0282938. doi: 10.1371/journal.pone.0282938 (PMC10957084; doi:10.1371/journal.pone.0282938)
Supplement: S1 Fig — (PDF) [file pone.0282938.s001.pdf]

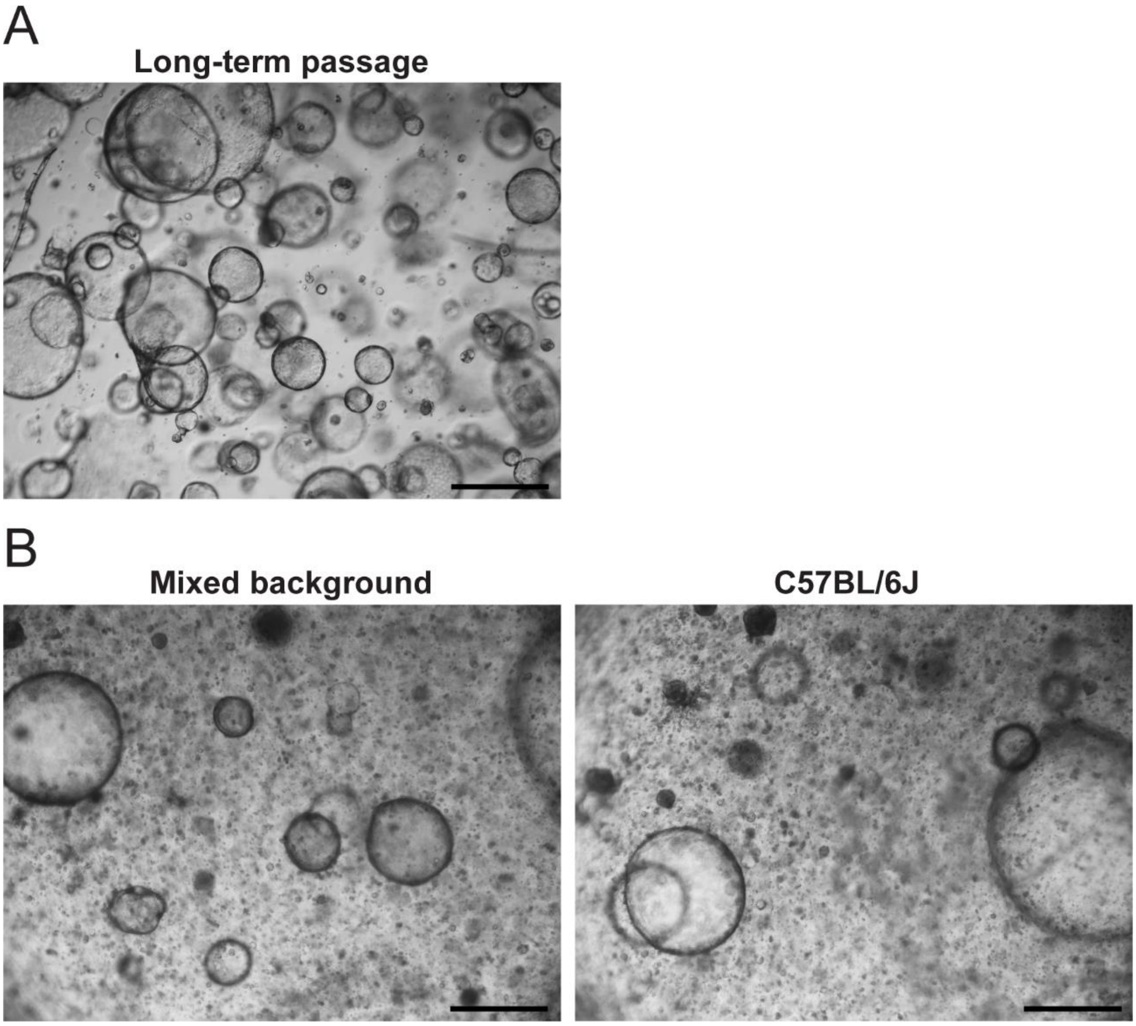

S1 Fig. Long-term culture of kidney tubuloids and establishing kidney tubuloids of different mouse backgrounds. (A) Brightfield image of an tubuloid culture, which was serially passaged for 3.5 months. (B) Brightfield image of a freshly seeded tubuloid culture from a mixed C57BL/6J and FVB/NJ background (left), and from the B57BL/6J background (right) mice. Data information: scale bars, 500 µm. Three independent replicates were examined.
