# Supplementary material for: Mice with renal-specific alterations of stem cell-associated signaling develop symptoms of chronic kidney disease but surprisingly no tumors
Source: PLoS One. 2024 Mar 21;19(3):e0282938. doi: 10.1371/journal.pone.0282938 (PMC10957084; doi:10.1371/journal.pone.0282938)
Supplement: Attachment — Submitted filename: Response to Reviewers.docx [file pone.0282938.s009.docx]

Berlin, November 16^th^, 2023

**Rebuttal letter**

Dear dr. Klymkowsky,

We would like to thank you and both reviewers for the efforts in assessing our revised manuscript. We are happy to hear that the manuscript will likely be accepted upon a few minor changes.

Editor:

*I agree with the reviewer 2 about the headings in the discussion, please removed - and address (again) their point about figure 2D. With these changes the ms. is likely to acceptable without re-review.*

Reviewer 2:

*The paper has now improved even if I think there is still the point 5 to address. Even after the author explanation, The Figure 2D remains misleading since even if the aim of Fig 2D was the functional confirmation of a high number of proliferating cells in tubuloids, the comparison between BrdU cells derived from organoids and EPCAM+ and adult kidney epithelial cells (control kidney) should be shown. I suggest eliminating Fig 2D and that to support the assertion that kidney tubuloids displayed enhanced proliferation showing a new graphic from their proteomic data showing the expression of proliferation markers Ki67, Pcna and Cyclin D1 in the three conditions.*

We have now fully addressed these points.

We look forward to hearing from you and would be glad to respond to any further questions or comments.

Yours sincerely,

Dr. Adam Myszczyszyn, first author

Prof. Dr. Walter Birchmeier, senior author
